# Cluster of factors related to metabolic changes in older individuals: a cross-sectional study

**DOI:** 10.1590/1516.3180.2023.0283.R1.30082024

**Published:** 2025-04-28

**Authors:** Clarice Alves dos Santos, Mariana Alves dos Santos, Manuela Alves dos Santos, Milena Fernandez Dias, Lélia Renata Carneiro Vasconcelos, Saulo Vasconcelos Rocha

**Affiliations:** I Department of Biological Sciences, Universidade Estadual do Sudoeste da Bahia (UESB), Jequié (BA), Brasil.; II Department of Health I, Universidade Estadual do Sudoeste da Bahia (UESB), Jequié (BA), Brasil.; III Department of Health I, Universidade Estadual do Sudoeste da Bahia (UESB), Jequié (BA), Brasil.; IV Department of Physical Education, Universidade de Brasília (UNB), Brasília (DF), Brasil.; V Department of Health II, Universidade Estadual do Sudoeste da Bahia (UESB), Jequié (BA), Brasil.; VI Department of Health II, Universidade Estadual do Sudoeste da Bahia (UESB), Jequié (BA), Brasil.

**Keywords:** Risk factors, Metabolic syndrome, Public health, Elderly, Elderly health, Cross-sectional studies, Metabolic changes

## Abstract

**BACKGROUND:**

Exposure to multiple risk factors related to metabolic changes can negatively affect the health status of older individuals.

**OBJECTIVE:**

To investigate the clustering of factors related to metabolic changes in older individuals.

**DESIGN AND SETTING:**

This was a cross-sectional study involving 287 older individuals (≥ 60 years old) enrolled in the Family Health Strategy in the municipality of Ibicuí, state of Bahia.

**METHODS:**

Factors associated with metabolic changes were abdominal obesity, self-reported diabetes, high blood pressure, sedentary behavior, and physical inactivity. Clustering was defined by an observed-to-expected prevalence (O/E) ratio greater than 1.20. The association between these factors was analyzed using multiple logistic regression.

**RESULTS:**

A total of seven clusters were identified with a predominance of diabetes, hypertension, sedentary behavior, and abdominal obesity (O/E = 2.28). Older adults were more likely to present with physical inactivity, diabetes, blood pressure, and sedentary behavior simultaneously (Odds Ratio [OR] = 7.78; 95% confidence interval [CI] = 1.25–48.42). Negative health perception was associated with the combination of high blood pressure, sedentary behavior, and abdominal obesity (OR = 0.23; 95%CI = 0.25–0.92); female sex with the cluster of physical inactivity and abdominal obesity (OR = 0.12; 95%CI = 0.04–0.35); and the occurrence of physical inactivity without the presence of other factors (OR = 3.87; 95%CI = 1.66–8.99).

**CONCLUSIONS:**

The combination of risk factors related to metabolic changes represents a greater probability of health problems than individual factors. Therefore, investigating the association between these factors will help in planning targeted interventions.

## INTRODUCTION

Metabolic changes, including abdominal obesity, hyperglycemia, dyslipidemia, and high blood pressure, are associated with an increased risk of diseases such as type 2 diabetes and cardiovascular disease, as well as an increase in overall mortality.^1,2^ In addition, these changes can lead to the progression of other clinical complications, such as cancer, gout, nonalcoholic fatty liver disease, polycystic ovary syndrome, sleep apnea syndrome, and dementia.^3^

The simultaneous occurrence of some of these alterations is referred to as metabolic syndrome (MS). This has been described as one of the main public health problems involving a cluster of visceral adiposity and a high risk of developing diabetes, cardiovascular disease, and mortality.^4^ One of the main contributors to its increasing prevalence is population aging, given that older individuals are routinely the most affected by the cardiovascular and metabolic risk factors that characterize MS.^5^

Studies have identified physical and functional decline,^6^ sedentary lifestyle,^7^ and physical inactivity^8,9^ as risk factors for MS and explained how these factors alter the body’s metabolic responses. Both sedentary behavior and physical inactivity contribute to deleterious metabolic effects, such as increased insulin resistance, changes in adipose tissue lipolysis, and mitochondrial pathway dysfunction. Moreover, these factors are correlated with the accumulation of visceral and hepatic fat in individuals at high risk of type 2 diabetes.^8,10^

Assuming that MS is a group of clinical changes influenced by lifestyle, sedentary lifestyle, unhealthy eating habits, stress and genetic factors and that their co-occurrence may be associated with an increased risk of developing diseases, cluster-type combinatorial analysis has emerged as an important tool for examining the relationship between multiple risk factors^11^. This type of analysis allows the identification of coexisting and aggregated different risk factors, enabling the evaluation of the most prevalent clusters.

Therefore, understanding common clusters of metabolic risk factors is relevant for the primary prevention of a specific target and the promotion of broader health policies,^12,13^ to apply more appropriate strategies for the management and prevention of health risks in a population.^14,15^ However, research on the clustering of metabolic risk factors among the Brazilian population is still incipient.

## OBJECTIVE

This study aimed to investigate the aggregation of factors related to metabolic changes in older individuals.

## METHODS

### Study design and sample

This cross-sectional study, based on data from the project titled “Monitoring the Health Conditions of the Elderly in a Small Municipality (MONIDI),” was conducted in the municipality of Ibicuí, state of Bahia, in 2014.

The target population of the current study was comprised of randomly selected older people of both sexes, aged 60 years or older, who were enrolled in the Family Health Strategy (FHS) of the municipality. The sample size was determined based on criteria for finite populations,^16^ adopting a significance level of 5%, 95% confidence interval (CI), and tolerable error of 3%. An additional 10% of individuals were included in the sample to compensate for possible losses and refusals, resulting in a total sample of 310 older individuals.

The exclusion criteria included being bedridden, having Alzheimer’s disease or other neurological and cognitive disorders, being diagnosed and reported by FHS professionals, the absence of any body segment, and conditions that would prevent participation in the physical fitness assessment or their ability to answer the questionnaire. Individuals who lacked information on any factors related to the metabolic changes used for the cluster analysis were excluded. After applying the eligibility criteria, 287 older adults were included in the final sample.

### Data collection and study variables

Data were collected using the Elderly Health Assessment Instrument tool.^17^ This instrument was used in the Family Health Units, along with a physical assessment conducted by a previously trained team. Older individuals were invited to visit the units on the day of data collection and were informed about the relevance and objectives of the study. Face-to-face interviews, physical tests, and anthropometric assessments were conducted.

For the current study, sociodemographic information and perceptions of health status were used as independent variables. These included sex (female or male), age (dichotomized into 60–79 years and ≥ 80 years), race/color (self-reported and categorized as white and non-white [black, brown, yellow, and indigenous]), marital status (with a partner or without a partner), income (< 1 minimum wage or ≥ 1 minimum wage), schooling (in completed years of study, categorized as literate and illiterate), and perceived health (dichotomized into positive [excellent, very good, and good] and negative [fair and poor]).

The five factors related to metabolic changes, which were used in the cluster analysis, are listed in **Table 1**.

**Table 1:** Description of the factors used in this study

Factors	Evaluation in research	Definition applied
Abdominal obesity (AO)	Anthropometric measurements of waist circumference (WC) were used to assess abdominal obesity (males ≥ 94; females ≥ 80).	Classified based on WHO recommended values for females and males.^18^
Self-reported diabetes (D)	Noted by the following question: Do you have any of the health problems listed below? Diabetes (yes or no)	Verified by an affirmative answer to the question.^19^
High blood pressure (BP)	Noted by the following question: Do you have any of the health problems listed below? High blood pressure (yes or no).	Verified by an affirmative answer to the question.^19^
Sedentary behavior (SB)	Assessed through questions taken from the International Physical Activity Questionnaire (IPAQ), a version adapted for older Brazilians.	Verified by calculating the average time spent in sedentary behavior.^20^
Physical inactivity (PI)	Assessed through the question: How would you rate your leisure-time physical activity?	Those who reported not practicing physical activity (light, moderate, or intense) in their free time were considered insufficiently active in their leisure time.^21^

WHO = World Health Organization.

### Data analysis

Descriptive statistics, including absolute and relative frequencies, were used in the data analysis to characterize behaviors related to metabolic changes based on the described variables. For the cluster analysis, the joint probability of these behaviors was calculated. The presence of clustering was verified by the ratio of observed (O) to expected (E) prevalence, with clusters defined as combinations where the O/E ratio was greater than 1.20.^22^

Logistic regression analysis was used to examine the associations between each combination of behaviors and independent variables. Combinations with the highest observed prevalence and O/E ratios greater than 1.20 were included in the adjustment models.

To identify the variables associated with each group, the odds ratio (OR) was adopted as an effect measure, with a 95%CI obtained through logistic regression analysis. All variables with a significance level of less than 0.20 in the bivariate analysis were included in the adjusted models. The multiple logistic regression analysis was conducted using models that included all preselected independent variables with a backward stepwise selection method. Analyses were performed using STATA software (version 14.0; StataCorp LLC, College Station, Texas, United States).

### Ethical aspects

This study adhered to the ethical principles of the Declaration of Helsinki by the World Medical Association and Resolution 466/2012 of the National Health Council. The research protocols were evaluated and approved on April 11, 2024, by the Human Research Ethics Committee of the Universidade Estadual do Sudoeste da Bahia (UESB) (CAAE: 22969013.0.0000.0055). All participants provided written informed consent before participation.

## RESULTS

The sociodemographic profiles of the participants and the prevalence of factors related to metabolic changes are shown in **Table 2**. The sample primarily consisted of older people aged 60 to 79 years (83.62%), females (54.01%), and those living without a partner (50.1%). Non-white races/colors were the most prevalent (69.3%) among the participants, and the majority were literate (56.1%).

**Table 2:** Prevalence of factors related to metabolic changes according to sociodemographic variables. MONIDI, Ibicuí/BA, 2014

Variables	Total	Physical inactivity		Diabetes		High blood pressure		Sedentary behavior		Abdominal obesity	
	n (%)	n (%)	P value	n (%)	P value	n (%)	P value	n (%)	P value	n (%)	P value
**Total**	287 (100%)	196 (68.29)		41 (14.29)		122 (42.51)		124 (43.21)		159 (55.40)	
**Age (years)**											
60–79	240 (83.62)	164 (68,33)	0.973	31 (12.92)	0.134	101 (42.08)	0.742	102 (42.50)	0.586	138 (57.50)	0.106
≥80	47 (16.38)	32 (69.09)		10 (21.28)		21 (44.68)		22 (46.81)		21 (44.68)	
**Sex**											
Female	155 (54.01)	110 (70.97)	0.291	18 (11.61)	0.161	67 (43.23)	0.790	64 (41.29)	0.478	120 (77.42)	**<0.001**
Male	132 (45.99)	86 (65.15)		23 (17.42)		55 (41.67)		60 (45.45)		39 (29.55)	
**Race/color (271)**											
White	83 (30.63)	51 (61.45)	0.131	15 (18.07)	0.202	31 (37.35)	0.260	35 (42.17)	0.762	49 (59.04)	0.569
Non-white	188 (69.37)	133 (70.74)		23 (12.23)		84 (44.68)		83 (44.15)		114 (55.32)	
**Marital status**											
Without a partner	144 (50.17)	92 (63.89)	0.108	19 (13.19)	0.596	65 (45.14)	0.366	71 (49.31)	**0.036**	80 (55.56)	0.958
With a partner	143 (49.83)	104 (72.73)		22 (15.38)		57 (39.86)		53 (37.06)		79 (55.24)	
**Schooling**											
Literate	161 (56.10)	102 (63.35)	**0.042**	18 (11.18)	0.089	64 (39.75)	0.285	71 (44.10)	0.730	97 (60.25)	0.062
Illiterate	126 (43.90)	94 (74.60)		23 (18.25)		58 (46.03)		53 (42.06)		62 (49.21)	
**Perceived health**											
Positive	110 (38.33)	71 (64.55)	0.282	10 (9.09)	**0.047**	45 (40.91)	0.666	49 (44.55)	0.718	67 (60.91)	0.139
Negative	177 (61.67)	125 (70.62)		31 (17.51)		77 (43.50)		75 (42.37)		92 (51.98)	

n = number of individuals per variable; % = Percentage; P value = Pearson’s chi-square test.

The most common risk factors identified were physical inactivity (68.2%) and abdominal obesity (55.4%). Regarding the associations between isolated risk factors and sociodemographic characteristics, older individuals who were illiterate were significantly more inactive (P = 0.042), those who lived without a partner reported more time spent in sedentary behavior (P = 0.036), and females had a higher prevalence of abdominal obesity (P < 0.001). Additionally, there was a greater prevalence of negative health perceptions among participants with diabetes (P = 0.047) (**Table 2**).

Overall, seven combinations were observed with an O/E value greater than 1.20. The combination of diabetes (D), high blood pressure (BP), sedentary behavior (SB), and abdominal obesity (AO) was most prevalent (O/E = 2.28; 95%CI = 2.14–2.41) (**Table 3**).

**Table 3:** Prevalence of cluster of factors related to metabolic changes in older individuals. MONIDI, Ibicuí/BA, 2014

Risk factors	PI	D	BP	SB	AO	O(%)	O/E	95%CI
5	+	+	+	+	+	1.05	1.06	(0.88–1.23)
4	-	+	+	+	+	1.05	**2.28**	(2.14–2.41)
4	+	-	+	+	+	6.97	1.17	(1.01–1.33)
4	+	+	-	+	+	1.05	0.78	(0.60–0.96)
4	+	+	+	-	+	1.74	**1.33**	(1.15–1.51)
4	+	+	+	+	-	1.74	**2.18**	(2.01–2.34)
3	-	-	+	+	+	3.48	**1.26**	(1.08–1.44)
3	-	+	-	+	+	0.0	0.0	(-0.16–0.16)
3	-	+	+	-	+	0.70	1.16	(1.00–1.31)
3	-	+	+	+	-	0.35	0.94	(0.83–1.06)
3	+	-	-	+	+	7.32	0.91	(0.77–1.05)
3	+	-	+	-	+	5.57	0.71	(0.57–0.85)
3	+	-	+	+	-	4.88	1.02	(0.85–1.19)
3	+	+	-	-	+	1.38	0.78	(0.60–0.96)
3	+	+	-	+	-	1.74	**1.61**	(1.43–1.79)
3	+	+	+	-	-	0.70	0.67	(0.49–0.84)
2	-	-	-	+	+	2.79	0.75	(0.57–0.92)
2	-	-	+	-	+	3.83	1.05	(0.88–1.23)
2	-	-	+	+	-	2.44	1.10	(0.91–1.28)
2	+	-	-	-	+	12.89	**1.22**	(1.11–1.33)
2	+	-	-	+	-	5.77	0.89	(0.73–1.05)
2	+	-	+	-	-	5.18	0.82	(0.67–0.98)
2	+	+	-	-	-	0.70	0.49	(0.31–0.67)
2	-	+	-	-	+	0.70	0.85	(0.68–1.02)
2	-	+	-	+	-	0.35	0.70	(0.56–0.84)
2	-	+	+	-	-	0.35	0.72	(0.58–0.86)
1	-	-	-	-	+	4.88	0.99	(0.82–1.16)
1	-	-	-	+	-	2.44	0.81	(0.63–0.99)
1	-	-	+	-	-	3.49	1.19	(1.01–1.37)
1	+	-	-	-	-	10.80	**1.27**	(1.13–1.40)
1	-	+	-	-	-	0.70	1.06	(0.90–1.22)
0	-	-	-	-	-	4.18	1.06	(0.88–1.23)

CI = confidence interval; + = presence of risk behavior; = absence of risk behavior; O = observed prevalence; O/E = relationship between observed and expected prevalence; PI = physical inactivity; D = diabetes; BP = high blood pressure; SB = sedentary behavior; AO = abdominal obesity.

The prevalence of the most frequent groups based on sociodemographic characteristics and health perceptions of older adults is shown in **Table 4**.

**Table 4:** Prevalence of groupings of factors related to metabolic changes according to sociodemographic characteristics and health perception of older individuals. MONIDI, Ibicuí/BA, 2014

Variables	D_BP_SB_AO (Cluster 1)	PI_D_BP_AO (Cluster 2)	PI_D_BP_SB (Cluster 3)	BP_SB_AO (Cluster 4)	PI_D_SB (Cluster 5)	PI_AO (Cluster 6)	PI (Cluster 7)
	n (%)	n (%)	n (%)	n (%)	n (%)	n (%)	n (%)
**Age (years)**							
60–79	3 (100.0)	5 (100.00)	2 (40.00)*	10 (100.00)	4 (80.00)	34 (91.89)	26 (83.87)
≥ 80	-	-	3 (60.00)	-	1 (20.00)	3 (8.11)	5 (16.13)
**Sex**							
Female	2 (66.67)	3 (60.00)	2 (40.00)	8 (80.00)	1 (20.00)	33 (89.9)*	8 (25.81)*
Male	1 (33.33)	2 (40.00)	3 (60.00)	2 (20.00)	4 (80.00)	4 (10.81)	23 (74.19)
**Race/color (282)**							
White	1 (33.33)	3 (60.00)	2 (66.67)	2 (20.00)	1 (20.00)	6 (17.14)	10 (34.48)
Non-white	2 (66.67)	2 (40.00)	1 (33.33)	8 (80.00)	4 (80.00)	29 (82.86)	19 (65.52)
**Marital status**							
Without a partner	2 (66.67)	1 (20.00)	4 (80.00)	7 (70.00)	2 (40.00)	18 (48.65)	13 (41.94)
With a partner	1 (33.33)	4 (80.00)	1 (20.00)	3 (30.00)	3 (60.00)	19 (51.35)	18 (58.06)
**Schooling**							
Literate	1 (33.33)	3 (60.00)	2 (40.00)	7 (70.00)	3 (60.00)	19 (51.35)	15 (48.39)
Illiterate	2 (66.67)	2 (40.00)	3 (60.00)	3 (30.00)	2 (40.00)	18 (48.65)	16 (51.61)
**Perceived health**							
Positive	-	1 (20.00)	2 (40.00)	7 (70.00)*	2 (40.00)	12 (32.43)	13 (41.94)
Negative	3 (100.00)	4 (80.00)	3 (60.00)	3 (30.00)	3 (60.00)	25 (67.57)	18 (58.06)

N = number of individuals per variable; % = Percentage;

*P value = < 0.05; PI = physical inactivity; D = diabetes; BP = high blood pressure; SB = sedentary behavior; AO = abdominal obesity.

When analyzing the combination of three simultaneous risk factors, a larger cluster was observed for the combination of physical inactivity, diabetes, and sedentary behavior (O/E = 1.61; 95%CI = 1.43–1.79). Among the combinations of two factors, the highest score was identified for the combination of physical inactivity and abdominal obesity (O/E = 1.22; 95%CI = 1.11–1.33). In the evaluation of the presence of only one of the factors without the presence of the others, physical inactivity had the highest prevalence (O/E = 1.27; 95%CI = 1.13–1.40) (**Table 3**).

In the multivariate analysis, associations were observed between the oldest age group and a combination of physical inactivity, diabetes, high blood pressure, and sedentary behavior (OR = 7.78; 95%CI = 1.25–48.42); negative perception of health was associated with a combination of high blood pressure, sedentary behavior, and abdominal obesity (OR = 0.23; 95%CI = 0.25–0.92); female sex was associated with a combination of physical inactivity and abdominal obesity (OR = 0.12; 95%CI = 0.04–0.35); and male sex was associated with a combination of physical inactivity without the presence of other risk factors (OR = 3.87; 95%CI = 1.66–8.99). These associations remained significant even after adjustments (**Table 5**).

**Table 5:** Results of multiple logistic regression between groups of factors related to metabolic changes and independent variables. MONIDI, Ibicuí/BA, 2014

Variables	PI_D_BP_SB	BP_SB_AO	PI_AO	PI
	OR (95%CI)	OR (95%CI)	OR (95%CI)	OR (95%CI)
Age (years)				
60–79	1	-	-	-
≥ 80	**7.78 (1.25–48.42)**	-	-	-
Sex				
Female	1	1	1	1
Male	1.47 (0.23–9.25)	0.25 (0.05–1.22)	**0.12 (0.04–0.36)**	**3.87 (1.66–8.99)**
Race/color (282)				
White	-	-	1	-
Non-white	-	-	1.34 (0.97–1.84)	-
Marital status				
Without partner	-	-	-	-
With partner	-	-	1.69 (0.79–3.61)	-
Schooling				
Literate	-	-	-	1
Illiterate	-	-	-	1.40 (0.65–3.01)
Perceived health				
Positive	-	1	-	-
Negative	-	**0.23 (0.25–0.92)**	-	-

CI = confidence interval; OR = odds ratio; PI = physical inactivity; D = diabetes; BP = high blood pressure; SB = sedentary behavior; AO = abdominal obesity.

## DISCUSSION

This study investigated the clustering of factors related to metabolic changes in older individuals. Of the 32 possible combinations identified in the cluster analysis, seven clusters whose risk factors did not occur independently in the population were verified. The co-occurrence of risk factors related to metabolic changes represents a greater probability of future cardiovascular complications than any single risk factor in isolation.^23^

Among the five factors related to metabolic changes, physical inactivity was present in five of the seven combinations with the highest observed-to-expected prevalence ratio. This result aligns with studies showing a consistent relationship between the increase in the prevalence of MS and insufficient levels of physical activity in Western populations.^7,24,25^ In older Brazilians, an increase in the level of physical activity was associated with a 33% lower chance of developing MS than inactivity.^26^

When analyzing the association between the groups with the highest prevalence and independent variables, the oldest individuals were seven times more likely to simultaneously present four of the five factors related to metabolic changes: physical inactivity, diabetes, arterial hypertension, and sedentary behavior. Previous findings reinforce this result and demonstrate how physical inactivity and sedentary behavior (both strongly related to obesity) are associated with an increased risk of several chronic diseases, including heart disease, diabetes, hypertension, osteoporosis, depression, and several types of cancers.^27,28^

Research involving 1,606 older participants in the baseline of the Bambuí Project showed a high prevalence of the primary diseases associated with MS, where 77.5% of the participants were solely hypertensive, 6.6% were solely diabetic, and 15.9% were both hypertensive and diabetic.^29^ This high prevalence can be attributed to increased exposure to risk factors such as physical inactivity, sedentary behavior, and obesity with aging, representing an important public health problem in Brazil.

Sedentary behavior establishes a positive relationship between risks and the health of older individuals. Time spent watching television and total sitting time greater than 2 h/day were associated with an increased relative risk of MS^30^ and increased risk of obesity.^31^ Individuals who spend more time in a sitting and/or inclined position (> 5 h/day) were 4.5 and 2.9 times more likely to have diabetes mellitus and dyslipidemia, respectively, compared with those with lower levels of sedentary behavior.^32^

The prevalence of the concomitant occurrence of multiple factors related to metabolic changes in the older population has rarely been described in national literature. A study involving patients with diabetes and hypertension monitored by the FHS in the state of Pernambuco reported that older adults (> 60 years) were more likely to spend 6 h/day or more sitting compared with those in the younger age group (20–59 years old).^33^

In this study, older adults with a negative perception of their health were more likely to present with high blood pressure, sedentary behavior, and abdominal obesity. These results align with findings from a previous study conducted on the Chilean national population, which indicated that a combination of physical activity and minimal time spent on behaviors related to sitting proved to be beneficial as a marker of adiposity and cardiometabolic health. Data from this study suggest that increased physical activity in people categorized as highly sedentary can help mitigate cardiometabolic risk.^34^

Both high blood pressure and abdominal obesity are associated with a higher risk of mortality from cardiovascular diseases.^26,35^ In Brazil, high BP affects more than 60% of the older population, contributing directly or indirectly to 50% of deaths from cardiovascular diseases, and shows a direct and linear association with aging.^26^

Studies have indicated a statistically significant association between sedentary behavior and abdominal obesity.^36,37^ A possible hypothesis for this association is that sedentary behavior promotes lower energy expenditure, replaces daily activities—even light intensity—and stimulates the consumption of high-calorie foods, which can result in greater adiposity.^38,39^ However, the combined association between these two factors and high blood pressure requires further investigation.

A survey conducted among climacteric females showed that abdominal obesity was associated with a sedentary lifestyle, elevated total cholesterol levels, and high blood pressure.^40^ The association between hypertension and abdominal obesity was observed in a 22-year Chinese cohort study, which demonstrated that body mass index and waist circumference significantly predicted the development of hypertension.^41^

However, the mechanism underlying the association between abdominal obesity and hypertension remains unclear. Nevertheless, the increase in adipose tissue likely leads to the release of a variety of adipokines linked to decreased production and utilization of nitric oxide, which plays important roles in regulating vascular tone and suppressing vascular smooth muscle cell proliferation. Therefore, a reduction in the effects of nitric oxide is associated with endothelial dysfunction and high blood pressure.^41,42,43^

In the current study, the simultaneous occurrence of physical inactivity and abdominal obesity was higher in females than in males. However, when evaluating the behavior of physical inactivity alone, males exhibited a 3.87 times greater probability of being physically inactive than females. These results suggest that females had a lower probability of being physically inactive when considered independently.

Physical inactivity and abdominal adiposity are associated with persistent low-grade systemic inflammation. A previous study highlights a probable link between these factors and a “sedentary-vicious cycle,” where physical inactivity leads to chronic inflammation induced by the accumulation of visceral fat and is commonly accompanied by fatigue and loss of muscle mass, which proportionally affects the ability to engage in physical activity.^44^

Our results reinforce the concept of distinguishing the clinical expression of metabolic changes according to sex, where the higher prevalence of physical inactivity and abdominal obesity among females may be related to postmenopausal status.^40,45^ A study conducted with climacteric females strengthened this relationship, demonstrating a higher prevalence of abdominal obesity in physically inactive females.^40^

Changes in menopause-related sex hormone levels have an additional negative impact on metabolic health.^46^ These changes are associated with changes in fat distribution patterns, visceral accumulation, and increased waist circumference^47,48^ and changes in metabolic biomarkers^49^ and decreased muscle mass.^50^ Consequently, menopause has a strong potential to contribute to the development or worsening of MS in females.^51^

A study showed that females have a higher percentage of barriers to engaging in physical activity than males.^52^ The barriers are socially constructed sex roles, the burden of managing the home, difficulty in accessing physical activity spaces and leisure opportunities, violence in public spaces, and the higher prevalence of comorbidities in females. These factors may explain the higher prevalence of physical inactivity in this group.^53,54,55,56^

The limitation of this study is that the information on self-reported sedentary behavior and physical activity, although a widely used strategy in epidemiological investigations, may be susceptible to memory bias. However, this study evaluated many older adults living in a region characterized by low education and income and insufficient access to health services. To the best of our knowledge, this is the first study to investigate the combined effects of multiple factors related to metabolic changes in a sample of older individuals from Bahia.

## CONCLUSION

The grouping of risk factors related to metabolic changes was associated with a greater risk of future cardiovascular problems than any individual risk factor. Therefore, this study is important, as its findings will help in planning interventions targeting the most prevalent combinations. Further studies using the same methodological approach could enhance the knowledge of common risk clusters in this population. Additionally, this study emphasizes the importance of promoting increased physical activity among the older population and reducing sedentary time through public health policies as strategies for reducing cardiometabolic risk.
